# Effects of redox cycling compounds on DT diaphorase activity in the liver of rainbow trout (*Oncorhynchus mykiss*)

**DOI:** 10.1186/1476-5926-4-4

**Published:** 2005-05-04

**Authors:** Joachim Sturve, Eiríkur Stephensen, Lars Förlin

**Affiliations:** 1 Department of Zoology, Zoophysiology, Göteborg University, Box 463, 405 30, Göteborg, Sweden

## Abstract

**Background:**

DT diaphorase (DTD; NAD(P)H:quinone oxidoreductase; EC 1.6.99.2) catalyses the two electron reduction of quinones, thus preventing redox cycling and consequently quinone dependent production of reactive oxygen species. In rat and mouse, a wide range of chemicals including polyaromatic hydrocarbons, azo dyes and quinones induces DTD. Bifunctional compounds, such as β-naphthoflavone (β-NF) and benzo(a)pyrene (B(a)P), induce DTD together with CYP1A and phase II enzymes by a mechanism involving the aryl hydrocarbon receptor (AHR). Monofunctional induction of DTD is mediated through the antioxidant response element and does not lead to the induction of AHR dependent enzymes, such as CYP1A. The aim of this study was to investigate the effects of prooxidants (both bifunctional and monofunctional) on the activity of hepatic DTD in rainbow trout (*Oncorhynchus mykiss*) in order to evaluate DTD suitability as a biomarker. We also investigated the effect of β-NF on hepatic DTD activity in perch (*Perca fluviatilis*), shorthorn sculpin (*Myoxocephalus scorpius*), eelpout (*Zoarces viviparus*), brown trout (*Salmo trutta*) and carp (*Cyprinus carpio*). In addition, the effect of short term exposure to prooxidants on catalase activity was investigated.

**Results:**

In rainbow trout, hepatic DTD activity is induced by the bifunctional AHR agonists β-NF and B(a)P and the monofunctional inducers naphthazarin, menadione and paraquat. Although exposure to both B(a)P and β-NF led to a strong 7-ethoxyresorufin-O-deethylase (EROD) induction, none of the monofunctional compounds affected the rainbow trout EROD activity. DTD was not induced by β-NF in any of the other fish species. Much higher DTD activities were observed in rainbow trout compared to the other fish species. Catalase activity was less responsive to short term exposure to prooxidants compared to DTD.

**Conclusion:**

Since rainbow trout hepatic DTD activity is inducible by both monofunctional and bifunctional inducers, it is suggested that rainbow trout DTD may be regulated by the same mechanisms, as in mammals. The fact that DTD is inducible in rainbow trout suggests that the enzyme may be suitable as a part of a biomarker battery when rainbow trout is used in environmental studies. It appears as if DTD activity in rainbow trout is higher and inducible compared to the other fish species studied.

## Background

The aquatic environment is exposed to a great number of pollutants. Effluents from industries and sewage treatment plants as well as drainage from urban and agricultural areas contain pollutants that may damage aquatic life. A large part of these compounds exert their toxic effect by generating reactive oxygen species (ROS), causing oxidative stress [1]. Compounds such as quinones, certain polycyclic aromatic hydrocarbons (PAH) metabolites and bipyridils generate ROS through their ability to redox cycle, a process where an enzymatic one electron reduction of the parent compound is followed by an autooxidation in the presence of molecular oxygen [2]. In this reaction, the oxygen will be reduced to a superoxide ion that can lead to the formation of other ROS such as hydrogen peroxide (H_2_O_2_) and hydroxyl radicals [3]. ROS causes cell injury by oxidizing lipids, proteins and DNA leading to membrane damage, enzyme malfunction and/or tumor formation. The cell has evolved an antioxidant defense system consisting of antioxidant enzymes and molecules as a defense against oxidative damage. Failure of the antioxidant system to counteract ROS mediated damage, either due to an increased ROS production or a malfunctioning antioxidant defense, will lead to a state of oxidative stress with concomitant oxidative damage [3].

Responses to xenobiotics, including molecular or biochemical changes or cellular damage are used as biomarkers of exposure or injurious effects [4]. Several field studies show changes in antioxidant enzyme activities and levels of antioxidant molecules, as well as oxidative damage, in fish from areas supposedly exposed to prooxidants [1,5,6]. Both short term and heritable tolerance of killifish (*Fundulus heteroclitus*) to toxic sediments in the Elisabeth river (VA, USA) was suggested to be partly due to an upregulation of the antioxidant defence system [7].

The antioxidant defense system is generally less responsive to xenobiotics compared to other biomarkers such as the cytochrome P4501A (CYP1A) mediated 7-ethoxyresorufin-O-deethylase (EROD) activity [4]. Despite this fact, and since oxidative stress is an important mechanism in the pathology of fish, it would be of interest to establish new oxidative stress biomarkers in aquatic organisms. Elevated rates of idiopathic lesions and neoplasia in fish from polluted sites were suggested to be related to pollutant induced oxidative stress [2]. DT diaphorase (DTD; NAD(P)H:quinone oxidoreductase; NQO1; EC 1.6.99.2) was proposed as a biomarker for quinones in the aquatic environment [8]. Quinones are of interest due to their widespread occurrence in the environment, both naturally as metabolites in plants as well as environmental contaminants, such as quinonoid metabolites derived from benzene and polyaromatic hydrocarbons [9-11].

DTD is a mainly cytosolic flavoenzyme that catalyzes the two-electron reduction of quinones into hydroquinones, thus counteracting redox cycling of prooxidants. Hydroquinones are more stable and less likely to undergo autooxidation [12-15]. DTD is characterized by its ability to utilize both NADH and NADPH as electron donors and to be inhibited by the anticoagulant dicoumarol [16]. In mammals, DTD can be induced by monofunctional and bifunctional inducers together with other phase II enzymes, such as glutathione S-transferases (GST) [17,18]. In mouse, the two regulatory elements ARE (antioxidant responsive element) and XRE (xenobiotic responsive element) have been found on the 5' promoter region of the NQO1 gene, and the XRE shows significant homology to the CYP1A1 XRE [19]. Monofunctional DTD induction is mediated through the ARE, whereas bifunctional compounds can induce DTD activity by two different mechanisms: (i) directly via the aryl hydrocarbon receptor (AHR) acting on the XRE on the NQO1 gene; or (ii) through electrophilic metabolites from the CYP1A1 phase 1 reaction acting on the ARE [18]. Bifunctional inducers consist of large planar aromatic compounds, such as PAHs, whereas monofunctional inducers are a diverse group of chemicals with a majority containing, or acquiring by metabolism, a Michael reaction acceptor structure [20]. Although DTD has been proposed as a biomarker for quinones and redox cycling compounds in fish [8], few studies address the effects of prooxidants on DTD in fish.

The aim of the present study was to study the effect of the monofunctional inducers paraquat (PQ), menadione (MN) and naphtazarin (5,8-dihydroxy-1,4-naphthoquinone; DHNQ), and of the bifunctional inducers β-naphthoflavone (β-NF) and benzo(a)pyrene (B(a)P), on the catalytic activity of DTD in rainbow trout (*Oncorhynchus mykiss*) liver. The aim was also to investigate the suitability of rainbow trout DTD as a biomarker for redox cycling compounds. β-NF, which proved to be a potent inducer of DTD activity in rainbow trout, was also chosen to study the inducibility of DTD in other fish species used as sentinel species in monitoring studies. These fishes were the perch (*Perca fluviatilis*), shorthorn sculpin (*Myoxocephalus scorpius*), eelpout (*Zoarces viviparus*), brown trout (*Salmo trutta*), and carp (*Cyprinus carpio*). In addition, we studied the effects of prooxidants on the activities of catalase and EROD. Catalase metabolizes H_2_O_2 _into molecular oxygen and water [21] and EROD reflects the catalytic activity of the phase I detoxifying enzyme CYP1A [22].

## Results

### DT diaphorase activity

All studied compounds, both bifunctional (β-NF and B(a)P) and monofunctional (MN, PQ and DHNQ), caused significant increases in hepatic DTD activity in rainbow trout (Table 1). DTD activity increased in all β-NF exposed groups, although the increase was only significant in the group exposed to the high dose (15 mg Kg^-1^) and after 5 days (Table 1). Treatment with a high B(a)P dose (15 mg Kg^-1^) also caused increased DTD activity after 5 days (Table 1).

**Table 1:** Hepatic DT-diaphorase, EROD and catalase activities in rainbow trout exposed to benzo(a)pyrene (B(a)P), β-naphthoflavone (β-NF), naphthazarin (DHNQ), menadione (MN), or paraquat (PQ), for 2 and 5 days.

	**Dose **(mg Kg^-1^)	**DT-diaphorase **nmol/(min × mg protein)		**EROD **pmol/(min × mg protein)		**Catalase **mmol/(min × mg protein)	
		2 days	5 days	2 days	5 days	2 days	5 days

**B(a)P**	**0**	49.7 (9.4)	51.4 (20.3)	7.7 (3.7)	4.9 (1.5)	278 (52)	327 (33)
	**5**	58.3 (12.6)	59.4 (11.1)	159.3 (76.1)^a^	237.6 (146)^a^	334 (128)	398 (126)
	**15**	38.8 (16.1)	74.4 (15.8)^ab^	323.4 (280)^a^	491.8 (220)^a^	376 (96)	357 (114)
**β-NF**	**0**	50.5 (11.9)	54.0 (9.0)	8.5 (3.3)	7.7 (6.7)	297 (86)	247 (69)
	**5**	69.2 (16.3)	77.4 (19.8)	896.5 (156)^a^	943.3 (327)^a^	268 (93)	182 (75)
	**15**	72.1 (28.1)	101.7 (42.1)^a^	832.5 (399)^a^	877.3 (340)^a^	334 (131)	226 (95)
**DHNQ**	**0**	38.2 (5.3)	30.2 (7.6)	5.1 (3.2)	3.2 (1.8)	256 (103)	261 (171)
	**1**	45.3 (14.6)	40.4 (5.7)^a^	3.6 (1.9)	4.0 (3.9)	360 (132)	311 (152)
	**3**	41.7 (8.7)	48.3 (8.0)^a^	3.2 (1.6)	0.8 (0.5)^ab^	261 (69)	320 (64)
**MN**	**0**	40.7 (3.4)	47.0 (7.6)	7.3 (8.8)	10.0 (7.4)	144 (65)	204 (97)
	**5**	54.0 (9.4)^a^	65.0 (13.2)^a^	n.m.	6.6 (6.2)	136 (37)	215 (47)^b^
	**15**	35.7 (6.7)	51.8 (6.4)	5.5 (2.1)	5.2 (2.5)	127 (38)	158 (65)
**PQ**	**0**	47.2 (11.7)	46.9 (7.6)	5.5 (3.9)	2.2 (1.1)	245 (73)	248 (97)
	**3**	53.0 (8.3)	71.0 (6.0)^a^	4.3 (2.4)	1.4 (0.8)^b^	229 (47)	348 (55)^ab^
	**10**	43.7 (6.0)	61.8 (20.4)^a^	3.9 (1.1)	2.7 (1.3)	155 (23)^a^	171 (37)^a^

Note: Values (n = 7) are presented as: mean (standard deviation); n.m. = not measured. ^a^Significantly different from control – *p *< 0.05; ^b^Significantly different from day 2 – *p *< 0.05.

Among the monofunctional inducers studied MN was the only one to cause a significant increase in DTD activity after 2 days (Table 1). After 2 days exposure to the low dose (5 mg Kg^-1^) of MN, rainbow trout displayed a significant increase in DTD activity, whereas no change was observed in the group exposed to a high dose (15 mg Kg^-1^) of MN. The same pattern was observed after 5 days exposure with a significant increase in DTD activity in the low dose (5 mg Kg^-1^) group and no increase in DTD activity in the group exposed to a high dose (15 mg Kg^-1^) of MN. The fish exposed to PQ showed the same trend as the MN exposed fish, with lower DTD activity in the high dose groups compared to the low dose groups. After 2 days exposure, DTD activity increased in the group treated with a low dose of PQ (3 mg Kg^-1^) but the difference was not statistically significant. After 5 days treatment, both the low and the high dose groups (3 and 10 mg Kg^-1^) displayed significantly higher DTD activity compared to the control group, even though the high dose group displayed lower DTD activity than the low dose group (Table 1). DHNQ treatment led to an increase in DTD activity in both the low dose (1 mg Kg^-1^) and the high dose (3 mg Kg^-1^) groups, after 5 days exposure (Table 1).

### EROD activity

EROD activity was significantly and strongly increased in all groups treated with β-NF and B(a)P (both low and high dose and after 2 and 5 days) (Table 1). The monofunctional inducers PQ and MN treatment did not affect EROD activity, whereas the group exposed to the high dose of DHNQ (15 mg Kg^-1^) displayed a significant reduction in EROD activity after 5 days exposure (Table 1).

### Catalase activity

PQ caused a significant decrease in catalase activity in the high dose groups (after both 2 and 5 days exposure) and a significant increase in the low dose group after 5 days exposure (Tab. 1). B(a)P, β-NF, MN and DHNQ caused no statistically significant effects on the catalase activity (Table 1).

### Comparison of DTD activity in different fish species

In contrast to rainbow trout, DTD activity did not increase in perch, carp, brown trout, eelpout or shorthorn sculpin treated with a high dose (15 mg Kg^-1^) of β-NF for 5 days. The DTD activity in these fishes was considerably lower compared to DTD activity in rainbow trout (Table 2).

**Table 2:** DT diaphorase activity in rainbow trout, brown trout, perch, carp, eelpout and shorthorn sculpin exposed to 15 mg Kg^-1 ^of β-naphthoflavone (β-NF) for 5 days.

	**DT diaphorase **nmol/(min × mg protein)	
	**Control**	**β-NF**

**Rainbow trout**	54.0 (9.0)	101.7 (42.1)^a^
**Brown trout**	14.9 (4.7)	11.1 (5.5)
**Perch**	4.7 (2.7)	6.3 (2.8)
**Carp**	5.2 (2.4)	4.0 (1.3)
**Eelpout**	3.2 (1.4)	2.8 (2.0)
**Shorthorn sculpin**	6.0 (2.8)	4.5 (2.8)

Note: Values are presented as mean (standard deviation); n = 7 for rainbow trout, and n = 6 for the additional fish species studied.

^a^Significantly different from control; *p *< 0.05.

## Discussion

The effects of β-NF and B(a)P on cellular defense systems have been extensively studied in fish, including rainbow trout. Both compounds have proved to be potent AHR agonists inducing enzymes in the CYP system, especially CYP1A [22]. Most studies in fish address effects of these compounds on the CYP system and relatively few have investigated effects on oxidative stress parameters. Stephensen *et al. *[23] demonstrated, in a short term study in rainbow trout liver, that treatment with 15 mg Kg^-1 ^of β-NF caused a moderate increase in cytosolic glutathione reductase (GR) activity and a decrease in the cytosolic GST activity. Longer exposure to a higher dose of β-NF (50 mg Kg^-1 ^for 14 days) increases GST activity in rainbow trout [24]. Regoli *et al. *[25] showed that both β-NF and B(a)P increased GR activity in European eel (*Anguilla anguilla*) liver, whereas both compounds suppressed GST and catalase activities, indicating an increase in oxidative stress caused by these two AHR agonists. Some AHR agonists can also induce DTD activity in rainbow trout. Förlin *et al. *[26] observed in a long term study of rainbow trout that polychlorinated biphenyls (PCB) and 3-methylcholanthrene (3-MC) caused an increase in DTD activity. It has previously been demonstrated that also β-NF induces DTD activity in rainbow trout [27]. In the present study, both β-NF and B(a)P caused an increase in hepatic DTD activity in rainbow trout. β-NF and B(a)P also caused a strong increase in the CYP1A mediated EROD activity in all exposed groups indicating strong AHR activation. The fact that exposure to the bifunctional inducers β-NF and B(a)P induced both DTD and CYP1A activities implies that the DTD induction can be mediated through activation of the AHR. In mammals, it has been demonstrated that both β-NF and B(a)P induce DTD activity [28,29]. Both compounds are classified as bifunctional since they induce DTD activity (via a mechanism involving the AHR) and have electrophilic metabolites that induce DTD activity (via the ARE) [18]. Therefore, the results obtained in this study imply that rainbow trout DTD activity can be induced by a mechanism involving the AHR, as in mammals. It is not yet known whether the promoter region of the rainbow trout DTD gene contains functional XRE or ARE. Thus, it is not clear whether the observed induction of DTD activity is mediated directly by the AHR, through the XRE, or it is caused by metabolites acting as monofunctional inducers through the ARE, or even whether both mechanism coexist. This should be investigated in future studies.

Relatively few studies address the effects of monofunctional inducers on oxidative stress parameters in fish. Stephensen *et al. *[23] investigated the effects on glutathione and glutathione dependent enzymes in rainbow trout exposed to the monofunctional inducers PQ, MN and DHNQ. All three compounds induced the catalytic activities of GR and GST, PQ being the most potent inducer. DHNQ also induced the activities of glutathione peroxidase and γ-glutamylcysteine synthetase, the rate-limiting enzyme in the glutathione synthesis. The same study also showed that glutathione levels were increased after exposure to PQ and MN. In the present study, the monofunctional inducers DHNQ, MN and PQ elevated hepatic DTD activity in rainbow trout, but none of those compounds induced EROD activity. This suggests that DTD activity in rainbow trout may be induced through an oxidant responsive element resembling the mammalian AREs. A study by Samson *et al. *[30] suggests that H_2_O_2 _dependent metallothionein induction in rainbow trout was mediated through ARE-like sequences on the metallothionein gene.

Our results show that high doses of prooxidants can lead to a decrease in DTD activity. Exposure to high doses of both PQ and MN resulted in lower DTD activities compared to exposure to low doses. Previous studies have shown that PQ is a potent redox cycler, strongly inducing GR and G6PDH activities [23,31]. The effect on G6PDH activity suggests that PQ exposure lead to the depletion of NADPH, a molecule crucial to the redox state in the cell and a cofactor in the catalytic activity of DTD and CYP1A enzymes. However, since DTD can also utilize NADH as an electron donor, the NADPH depletion should not affect DTD activity. The lower DTD activities in the rainbow trout exposed to high doses of MN and PQ, when compared to activities from animals exposed to lower doses, could be due to an overproduction of ROS; causing an oxidation dependent malfunction of the DTD enzyme. Our results also show that catalase activity was decreased after the exposure to a high dose of PQ. Catalase can be partially inhibited by the ROS superoxide [3] and it is possible that also DTD is affected by a similar mechanism. In contrast, exposure to a high dose of DHNQ led to a decrease in EROD activity and an increase in DTD activity in rainbow trout. As previously reported [32,33], the decrease in EROD activity could be due to ROS mediated inactivation of the CYP1A enzyme.

A great number of evidence shows that the main function of mammalian DTD is to protect the cell against harmful ROS production caused by redox cycling compounds [34]. Exposure to such compounds causes an increase in DTD activities in rodents [29]. Studies also demonstrate that a lack of DTD activity, either due to knockout of the DTD gene or by dicoumarol enzyme inhibition, causes an increase in quinone dependent toxicity [12,35]. The cells inability to break the redox cycling of compounds, such as quinones, increases the ROS generation that leads to increases in oxidative cellular damage, which may result in tissue malfunction. The monofunctional compounds included in this study exert their toxic effect through redox cycling, which causes ROS production [23]. Metabolites from the bifunctional compounds may also cause ROS production through redox cycling [23]. The increase in hepatic DTD activity in rainbow trout by these compounds implies that DTD has a protective role also in this species. The increase of hepatic DTD activity in response to prooxidant exposure in rainbow trout may also suggest DTD activity as part of an oxidative stress biomarker battery. Cultivated rainbow trout are often used in fresh water caging studies for environmental biomonitoring. Biomarkers can be defined as specific biological changes resulting from exposure to chemicals. In order to qualify as an enzymatic biomarker, the enzyme needs to be induced or changed in a measurable way as a result to an exposure. This study shows that hepatic DTD activity in rainbow trout is inducible by prooxidants in short term exposure studies, suggesting that hepatic DTD may serve as a biomarker for prooxidants in rainbow trout. However, rainbow trout were exposed to high doses of the test compounds and the elevation of DTD activity was moderate (2-fold). Long-term studies with ecologically relevant doses would be necessary to fully evaluate the suitability of DTD as a biomarker.

Hepatic catalase activity is frequently used as a biomarker to assess oxidative stress in biomonitoring programs in the aquatic environment [36-38]. Catalase is mainly a peroxisomal enzyme, and it is thus possible that an elevation of catalase activity reflects peroxisomal proliferation rather than antioxidant defense. It has been shown that catalase activity positively correlates the number of peroxisomes in mice [39]. Though elevation in catalase activity is observed in field studies, few laboratory studies have reported increased catalase activities in fish exposed to prooxidants [4]. In the present study, only exposure to PQ and not to β(a)P, β-NF, DHNQ or MN affected catalase activity. This suggests that catalase is only slightly responsive to acute prooxidant exposure.

Among the fish species included in this study, rainbow trout was the only species that displayed inducible DTD activities. Treatment with 15 mg Kg^-1 ^of β-NF for five days doubled the DTD activity in rainbow trout liver cytosol, whereas no increase in DTD activity was observed in the other fish species receiving the same treatment, including the brown trout (*Salmo trutta*) that is more phylogenetically related to the rainbow trout. β-NF is believed to be rapidly metabolized by CYP1A enzymes and a dose of 15 mg Kg^-1 ^causes a high but not maximal induction of CYP1A activities in rainbow trout. Since different species differ in their sensitivity towards AHR agonists, 15 mg Kg^-1 ^was chosen as a moderate dose to initially screen the effects of a possible DTD inducer on fish species commonly used as sentinel ones in monitoring studies. This dose was chosen to avoid a too high dose capable of inhibiting DTD activity but also high enough for inducing DTD activity. Nevertheless, further studies should be conducted to investigate effects of increased number of doses and duration of exposures. On the other hand, studies in other fish species report low levels of DTD activity and inability of AHR agonists to induce DTD activity [27,40]. For example, wild sea bass (*Dicentrarachus labrax*) and dab (*Limanda limanda*) exposed to 3-MC displayed low DTD activities (ca. 4 and 3 nmol min^-1 ^mg^-1^, respectively, in control fish) and no DTD induction [27]. Pretti *et al. *[40] reported low DTD activities in gilthead seabream (*Sparus auratus*) (0.7 nmol min^-1 ^mg^-1 ^in control fish) and observed no elevation in DTD activity after exposing the fish to a high dose (50 mg Kg^-1^) of β-NF. Previous studies in rainbow trout exposed to the AHR agonist 3-MC and β-NF showed increased hepatic DTD activities [26,27].

Rainbow trout also displayed much higher DTD activities compared to the other fish species included in this study. When comparing the activities of different detoxification enzymes in different fish species, Förlin *et al. *[41] described a similar pattern with higher DTD activities in rainbow trout compared to other fish species. It was suggested that the observed species difference could be due to the presence of DTD inducing compounds in the commercial fish food, being rainbow trout the only cultivated fish species included in the study. This suggestion, however, is not fully supported by the fact that DTD activity was not induced in the other fish species treated with β-NF. Instead, it seems that the rainbow trout has higher natural basal levels of DTD activity compared to other fish species. It is also possible that rainbow trout express an inducible isoform of the enzyme, different from that found in other investigated fish species. The reason for this apparent species difference in relation to DTD is not known. It would be of interest to investigate whether the rainbow trout specific feature of DTD is natural or acquired. Stocks of rainbow trout have been cultivated for up to one hundred years and the species may have adapted to the special conditions in a fish farm. Higher basal levels of a more effective DTD enzyme could be one such adaptive response to prooxidants in commercial fish foods.

## Conclusion

Since rainbow trout DTD activity is inducible by both monofunctional and bifunctional inducers, we suggest that rainbow trout DTD gene expression may be regulated by the same mechanisms, as in mammals. The inducibility of rainbow trout hepatic DTD may also suggest that rainbow trout DTD have a protective role. The rainbow trout has a higher basal DTD activity compared to other fish species studied, and its activity is inducible. The fact that DTD activity is inducible in rainbow trout suggests that the enzyme may be suitable as a part of a biomarker battery; for example, in biomonitoring caging studies where cultivated rainbow trout are being used. It appears that DTD activity is not suitable as a biomarker in the other fish species investigated in this study, and that further studies are needed to elucidate the DTD response to prooxidants in these fish species.

## Methods

### Chemicals

β-naphthoflavone (β-NF) was obtained from Jansson Chimica and menadione (MN), paraquat (PQ), 5,8-Dihydroxy-1,4-naphthoquinone (naphthazarin; DHNQ) and benzo-a-pyrene (B(a)P) were obtained from Sigma (St. Louis, USA). 7-ethoxyresorufin, reduced β-nicotinamide adenine dinucleotide 2'-phosphate (NADPH), reduced β-nicotinamide adenine dinucleotide (NADH), bovine serum albumine (BSA) and 2,6-dichlorophenol indophenol (DCPIP) were obtained from Sigma (St. Louis, USA). Rhodamine B and H_2_O_2 _were obtained from MERCK (Darmstadt, Germany) and Dicoumarol from Aldrich (Milwaukee, USA). All other chemicals were of analytical grade.

### Fish

Cultured juvenile rainbow trout of both sexes, weighting 108 (SD = 24) g, were obtained from Antens fiskodling AB, a hatchery close to Göteborg. At least a week prior to the experiments, the fish were acclimatized to the laboratory conditions in 500 l tanks with filtered, aerated fresh water at 10°C in a flow-through system (5 l water/min). During the experiment, fish were kept in 50 l glass aquaria (7 fish per aquaria) with filtered, aerated water at 10°C, also in a flow-through system (0.5 l water/min). The fish were not fed and were exposed to a 12:12 h light/dark cycle.

Perch (*Perca fluviatilis*) weighing 6 (SD = 0.8) g, shorthorn sculpin (*Myoxocephalus scorpius*) weighing 153 (SD = 41) g, eelpout (*Zoarces viviparus*) weighing 35 (SD = 13) g, and brown trout (*Salmo trutta*) weighing 102 (SD = 23) g, were provided by local fishermen, and carp (*Cyprinus carpio*) weighing 155 (SD = 42) g was obtained from a local hatchery. Perch, brown trout and carp were treated as described for rainbow trout. Eelpout and shorthorn sculpin were kept in salt water (30‰), also under similar conditions as described for rainbow trout.

### Experimental design

At the start of the experiment, fish were injected intraperitoneally with the test compound dissolved in its respective carrier solution (peanut oil for β-NF, B(a)P, MN and DHNQ and 0.15 M KCl for PQ) or the carrier solution alone (0.5 ml/100 g fish). Rainbow trout were divided into three groups: one control group that received the carrier solution alone; one group that was exposed to a low dose of a single test compound (B(a)P, β-NF or MN 5 mg Kg^-1^, K DHNQ: 1 mg Kg^-1^, K PQ: 3 mg Kg^-1^); and, finally, one group that was exposed to a high dose of a single test compound (B(a)P, β-NF or MN 15 mg Kg^-1^, DHNQ, 3 mg Kg^-1^, PQ, 10 mg Kg^-1^). Doses were chosen relative to PQ toxicity data (manufacturers label, mammalian data) with the high PQ dose 10 times lower than recorded LD50 values. Since B(a)P, β-NF and MN are less toxic than PQ, these compounds were administered in higher doses. Due to DHNQ's higher toxicity we used lower doses of this compound. The exposure times were 2 and 5 days, and 7 fish were sampled per exposure group and time point.

Fish were killed with a sharp blow to the head and the weight and length recorded. Each fish was cut open and the liver excised, weighed and homogenized in Na^+^/K^+^-phosphate buffer (pH 7.4) containing 0.15 M KCl. The homogenate was centrifuged for 10 min at 700 g, and the supernatant recentrifuged for 20 min at 10,000 g. An aliquot of the supernatant (S9 fraction) was stored at -80°C for EROD measurements, and the rest of the supernatant centrifuged for 60 min at 105,000 g. Aliquots of the supernatant (cytosolic fraction) were stored at -80°C until analyzed. All subcellular preparation steps were performed at 4°C.

The additional fish species studied were treated with a high dose (15 mg Kg^-1^) for 5 days. The fish were exposed and sampled and the samples treated as described for rainbow trout. Six fish were sampled per exposure group.

### Biochemical assays

DT-diaphorase activity was measured in liver cytosolic fraction according to Ernster [13], modified and adapted to a microplate reader [42]. The reaction mixture contained 50 mM Tris-HCl (pH 7.6), 40 μM DCPIP and 0.3 mM NADH in a final volume of 210 μl. Ten μl of the sample were pipetted into microplate wells and the reaction was started by the addition of 200 μl of reaction mixture. Samples were measured with or without the addition of 0.1 mM dicoumarol, dissolved in 0.15 % NaOH. DTD activity was defined as dicoumarol inhibitable DCPIP reduction. Change in absorbance was monitored at 600 nm, and DTD activity calculated using the extinction coefficient for DCPIP (ε = 21 mM^-1^cm^-1^).

EROD activity was measured in liver S9 fraction according to the method described by Förlin *et al. *[43] using rhodamine as standard. The reaction mixture contained 0.1 M Na-phosphate buffer (pH 8.0), 0.5 μM ethoxyresorufin and 25–50 μl of sample in a final volume of 2 ml. The reaction was started with the addition of 10 μl 10 mM NADPH. The increase in fluorescence was monitored at 530 nm (excitation) and 585 (emission).

Catalase activity was measured in liver cytosol according to the method described by Aebi [21]. The reaction mixture contained 50 mM K-phosphate buffer (pH 6.5) and 50 mM H_2_O_2 _diluted in 80 mM K-phosphate buffer (pH 6.5). The mixture was incubated at 25°C, the baseline recorded, and the decrease in absorbance further recorded at 240 nm, after the addition of the sample. Catalase activity was calculated using the extinction coefficient for H_2_O_2 _(ε = 40 M^-1^cm^-1^).

Protein content was determined using the BCA Protein Assay Kit (Pierce, USA), with BSA as standard.

### Statistical analysis

Data were analyzed with one-way analysis of variance (ANOVA) and, following significant differences, with Newman-Keuls post hoc test. Levene's test was used to test for homogeneity of variances. Data displaying heterogeneity of variances were instead analysed using Kruskal-Wallis H test, followed by Dunnett's C test. When comparing only two groups, the Mann-Whitney U test was used. All statistical analyses were calculated using SPSS^® ^for Windows. The significance level (α) was set at 0.05. Data are presented as: mean (standard deviation).

## Authors' contributions

JS participated in the experimental design, fish exposure, sampling, performed most of the analysis and writing of the manuscript. ES participated in the experimental design, fish exposure and the sampling. LF rose funding and participated in the experimental design and writing of the manuscript. All authors read and approved the final manuscript.
